# TranSIC-Net: An End-to-End Transformer Network for OFDM Symbol Demodulation with Validation on DroneID Signals

**DOI:** 10.3390/s25206488

**Published:** 2025-10-21

**Authors:** Zhihong Wang, Zi-Xin Xu

**Affiliations:** College of Electronic Engineering, Chengdu University of Information Technology, Chengdu 610225, China; 19822961185@163.com

**Keywords:** deep learning, OFDM, Transformer, channel estimation, symbol decision, DroneID

## Abstract

Demodulating Orthogonal Frequency Division Multiplexing (OFDM) signals in complex wireless environments remains a fundamental challenge, especially when traditional receiver designs rely on explicit channel estimation under adverse conditions such as low signal-to-noise ratio (SNR) or carrier frequency offset (CFO). Motivated by practical challenges in decoding DroneID—a proprietary OFDM-like signaling format used by DJI drones with a nonstandard frame structure—we present TranSIC-Net, a Transformer-based end-to-end neural network that unifies channel estimation and symbol detection within a single architecture. Unlike conventional methods that treat these steps separately, TranSIC-Net implicitly learns channel dynamics from pilot patterns and exploits the attention mechanism to capture inter-subcarrier correlations. While initially developed to tackle the unique structure of DroneID, the model demonstrates strong generalizability: with minimal adaptation, it can be applied to a wide range of OFDM systems. Extensive evaluations on both synthetic OFDM waveforms and real-world unmanned aerial vehicle (UAV) signals show that TranSIC-Net consistently outperforms least-squares plus zero-forcing (LS+ZF) and leading deep learning baselines such as ProEsNet in terms of bit error rate (BER), estimation accuracy, and robustness—highlighting its effectiveness and flexibility in practical wireless communication scenarios.

## 1. Introduction

Orthogonal Frequency Division Multiplexing (OFDM) underpins modern wireless systems like Wi-Fi, LTE, and 5G due to its high spectral efficiency and robustness against multipath fading. By distributing data across orthogonal subcarriers, OFDM mitigates frequency-selective fading and simplifies equalization. However, reliable performance depends on accurate channel estimation, which is challenging under high mobility, carrier frequency offset (CFO), and low-SNR conditions [1,2,3].

With the rapid development of unmanned aerial vehicle (UAV) systems, commercial drones such as those produced by DJI have increasingly adopted proprietary wireless communication protocols. These protocols often employ OFDM-based broadcast signals, such as DroneID, which are proprietary but structured, making them a valuable source of information for UAV detection and identification. Efficient extraction of channel information and modulation symbols from such signals is critical for subsequent decoding and retrieval of UAV-related data [4,5]. Beyond detection and identification, UAV communication has also been studied in broader contexts such as vehicular networks and mobile video streaming, where task offloading and secure transmission are crucial for performance and safety [6,7].

Recent studies have applied deep learning to OFDM channel estimation and demodulation [8]. For example, Liu et al. [9] summarized classical methods and their limitations. Soltani et al. [10] cast channel estimation as image reconstruction, improving robustness at low SNR. Yi and Zhong [11] introduced joint channel estimation and detection frameworks. Residual and attention-based models, such as those by Gao et al. [12] and Yang et al. [13], further enhanced frequency-domain modeling and edge deployment. Other works addressed doubly selective channels and non-stationary conditions through joint time–frequency modeling [14], recursive inference [15], and subspace learning [16]. In parallel, sparsity-aware estimation techniques have gained attention in large-scale and hybrid-field communication systems. For example, Wang et al. [17] proposed a joint sparse Bayesian learning (SBL) framework that significantly enhances channel estimation accuracy by leveraging structural sparsity across antenna domains. Such approaches demonstrate the potential of model-driven sparsity exploitation as an alternative to conventional pilot-based estimation, motivating our exploration of fully data-driven end-to-end demodulation. Federated and adaptive learning methods have also been explored for low-complexity, privacy-preserving deployment [18,19].

Nonetheless, these methods largely embed deep models into conventional pipelines, limiting end-to-end learning—as in SCBiGNet [20], which still depends on preprocessing outputs like LS estimates. Moreover, most research targets standard protocols, while UAV-specific signals like DroneID pose unique challenges due to non-standard modulation and framing. Prior works focus mainly on detection or classification, with little on physical-layer demodulation or end-to-end symbol recovery.

These methods suffer from noise sensitivity, error propagation, and strong reliance on precise pilot estimates, while the decoupled channel estimation–equalization–demodulation architecture limits joint optimization and robustness.

To address these issues, we propose TranSIC-Net, a Transformer-based end-to-end demodulation network that outputs symbol probabilities directly from frequency-domain OFDM signals. Leveraging multi-head self-attention, it jointly exploits pilot structure and inter-symbol correlations, bypassing explicit channel estimation and enhancing performance under low-SNR and UAV-specific channel distortions.

TranSIC-Net consists of lightweight Transformer encoder blocks with learnable positional encodings and residual connections, followed by a shallow classifier, balancing performance and efficiency. Trained with a symbol-level classification loss, it directly infers modulation symbols from received signals.

We also evaluated TranSIC-Net on a real-world dataset of DJI DroneID signals subject to practical impairments such as carrier frequency offset (CFO), multipath fading, and additive noise. Compared to traditional LS/MMSE methods and the deep learning approach in [21], TranSIC-Net achieves lower bit error rates (BERs), particularly under low-SNR conditions (below 15 dB), demonstrating its practical effectiveness for UAV communication scenarios.Unlike prior Transformer-based models primarily designed for channel estimation or sequence modeling, TranSIC-Net directly maps frequency-domain OFDM symbols to symbol-level probabilities in an end-to-end manner. This eliminates the dependence on intermediate channel estimates while explicitly leveraging UAV-specific signal structures (e.g., DroneID framing). Furthermore, its lightweight design ensures practical feasibility for real-time UAV monitoring.

## 2. DroneID Signal Characteristics and Modeling

The DroneID signal is a proprietary wireless communication format developed by DJI, representing a specialized variant of Orthogonal Frequency Division Multiplexing (OFDM). As an OFDM-based signal, it inherits key properties such as high spectral efficiency, dense symbol packing, and resilience to multipath interference. Broadcast periodically in short frames across multiple frequency channels, its physical-layer design closely resembles general OFDM protocols. Although some parameters, such as pilot placement, subcarrier allocation, and modulation schemes, are customized for this protocol, the overall structure remains largely consistent with mainstream OFDM standards like Wi-Fi and LTE [22].

### 2.1. Structural Analysis of DroneID Signal

According to the study by Conner Bender [23], DJI DroneID signals are broadcast using the OcuSync protocol, employing frequency-hopping transmission over 13 frequency points in the 2.4 GHz and 5.8 GHz bands. During each hopping cycle, approximately 12–20 DroneID signals are transmitted per frequency point, with OFDM as the underlying modulation scheme.

In this work, each DroneID frame consists of nine OFDM symbols, while the fourth and sixth symbols serve as pilots, each embedding a Zadoff–Chu (ZC) sequence with distinct root indices. The remaining seven symbols carry QPSK-modulated data. Each symbol employs a 1024-point FFT, with active subcarriers ranging from indices k=212 to k=813 (excluding the DC subcarrier at k=512), totaling $N_{d}=600$ subcarriers used for data transmission. All data symbols are QPSK-modulated and normalized to unit magnitude in the complex plane.

Regarding cyclic prefix (CP) configuration, long CPs of length $L_{\mathrm{CP}}=80$ are used for the first and last symbols, while short CPs of length $L_{\mathrm{CP}}=72$ are used for the intermediate ones. After frequency-domain mapping, inverse FFT (IFFT) and CP insertion are applied to generate complete time-domain baseband frames.

### 2.2. Channel Modeling

To emulate realistic wireless propagation effects, the generated baseband signals are sequentially subjected to carrier frequency offset (CFO), multipath fading, and additive Gaussian noise, as illustrated below.

(1) Carrier Frequency Offset (CFO)

A CFO Δf (in Hz) is introduced to emulate the frequency mismatch between the transmitter and receiver oscillators. This offset introduces a phase rotation across OFDM symbols, modeled as

(1) $$s^{\prime }\left[n\right]=s\left[n\right]\;\cdot \;e^{j2\pi \Delta fn/f_{s}}$$

where s[n] is the transmitted time-domain signal, $s^{\prime }\left[n\right]$ is the CFO-distorted signal, and $f_{s}$ is the sampling rate.

(2) Multipath Rayleigh Fading

Next, the signal experiences multipath propagation characterized by a frequency-selective Rayleigh fading channel. It is modeled using a finite impulse response (FIR) filter:

(2) $$h=\left[h_{0},h_{1},\ldots ,h_{L-1}\right],\;h_{l}∼\mathrm{CN}\left(0,\frac{1}{L}\right)$$

(3) $$s^{\prime \prime }\left[n\right]=\sum_{l=0}^{L-1}h_{l}\;\cdot \;s^{\prime }\left[n-l\right]$$

where *L* denotes the number of channel taps, and each complex gain $h_{l}$ captures the amplitude and phase of the *l*-th propagation path.

(3) Additive White Gaussian Noise (AWGN)

Finally, the received signal is corrupted by complex Gaussian noise to simulate receiver thermal noise and interference:

(4) $$y\left[n\right]=s^{\prime \prime }\left[n\right]+w\left[n\right],\;w\left[n\right]∼\mathrm{CN}\left(0,\sigma^{2}\right)$$

where y[n] is the received signal and w[n] is zero-mean complex noise with variance $\sigma^{2}$.

### 2.3. DroneID Dataset Construction Process

This study builds the required training dataset by generating received time-domain signals and corresponding frequency-domain channel responses based on the frame structure and channel characteristics of DroneID signals. The complete data generation process includes bit mapping, frequency-domain symbol construction, OFDM modulation, channel distortion modeling, and data export as complex-valued sequences.

First, a bit sequence $b=[b_{0},b_{1},\ldots ,b_{N_{b}-1}]$ is generated and mapped to a QPSK symbol sequence $x=[x_{0},x_{1},\ldots ,x_{N_{d}-1}]$, where $N_{d}=600$. No scrambling or channel coding is applied to enable direct BER evaluation.

A frequency-domain vector $X=[X_{0},X_{1},\ldots ,X_{N-1}]$ is constructed, where N=1024 is the IFFT size. Nonzero elements are assigned to subcarriers in the index set $K_{\mathrm{data}}$:

(5) $$X_{k}=\left\{\begin{array}{cc} x_{i}, & k\in K_{\mathrm{data}}\\ 0, & \mathrm{otherwise} \end{array}\right.$$

The fourth and sixth OFDM symbols are designated as pilots and contain Zadoff–Chu (ZC) sequences:

(6) $$z\left[n\right]=e^{-j\frac{\pi rn(n+1)}{M}},\;n=0,1,\ldots ,M-1$$

where *M* is the ZC sequence length and *r* is the root index (e.g., r=600 or 147). The DC component is removed before frequency-domain embedding.

After constructing X, the time-domain symbol is obtained via IFFT:

(7) $$s_{n}=\frac{1}{N}\sum_{k=0}^{N-1}X_{k}\;\cdot \;e^{j2\pi kn/N},\;n=0,1,\ldots ,N-1$$

Cyclic prefixes of length $L_{\mathrm{CP}}$ are prepended to each OFDM symbol, and the entire frame is assembled and passed through the channel model to produce the received signal y[n].

**Figure 1:**
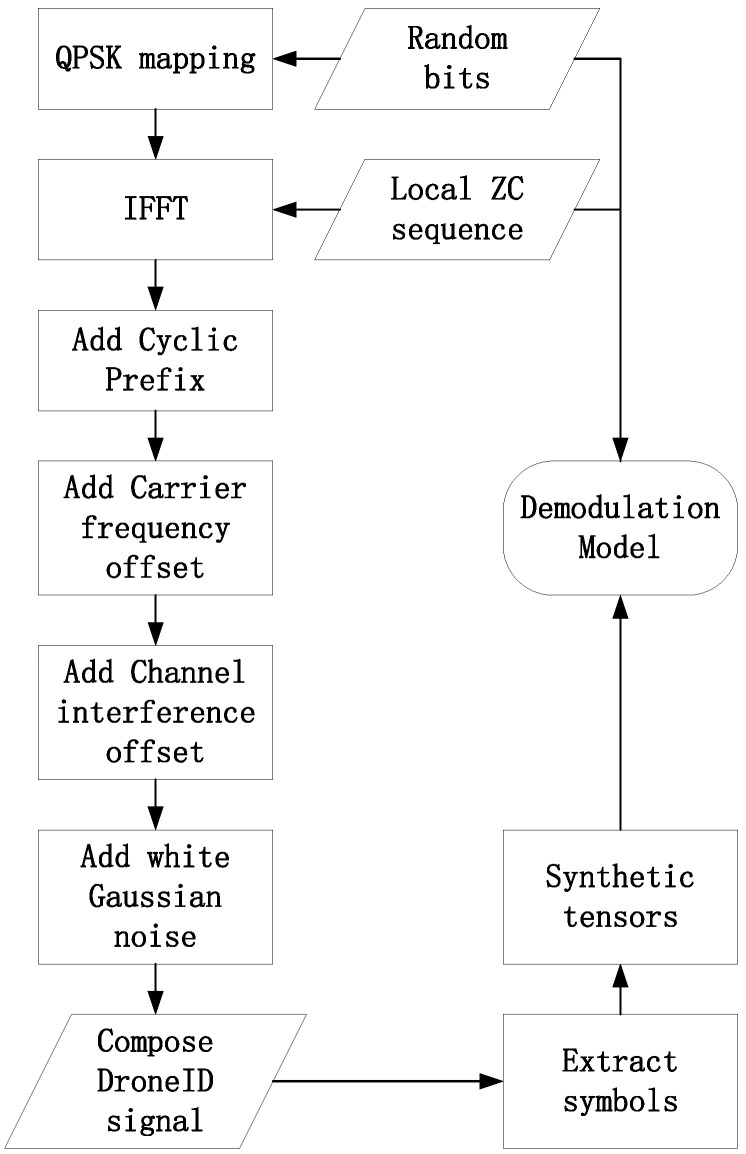
DroneID dataset generation pipeline.

As illustrated in Figure 1, the full process from bit generation to model input construction is depicted for the DroneID dataset.

The final outputs include

- Time-domain received signal y[n], saved as a complex-valued sequence;
- Corresponding bit sequence b for each frame, used as ground truth labels.

## 3. Deep Learning Model Design

**Figure 2:**
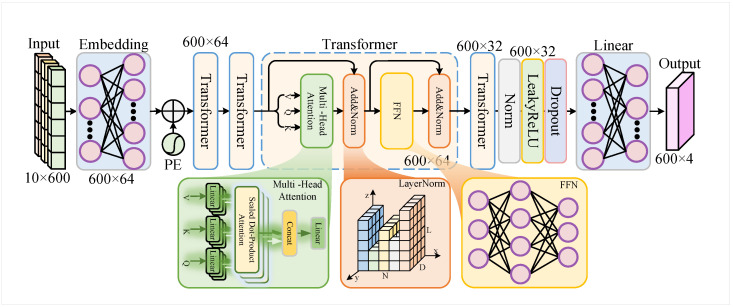
Architecture of TranSIC-Net.

To achieve reliable recovery of DroneID signals under practical channel conditions, this paper proposes an end-to-end deep neural network architecture called TranSIC-Net (Transformer-based Symbol and Implicit Channel estimation Network), tailored for frequency-domain OFDM symbols. Unlike traditional demodulation schemes that rely on explicit pilot-based channel estimation followed by equalization and symbol detection, the proposed method directly outputs the posterior probabilities of QPSK symbols on each data subcarrier from multi-symbol frequency-domain tensors containing both pilot and data symbols. Effectively, this model serves as a channel-aware symbol classifier, as illustrated in Figure 2, which presents the overall architecture of TranSIC-Net.

### 3.1. Modeling Task and Input–Output Format

The model estimates the QPSK symbol class on each subcarrier from the frequency-domain observations of multiple OFDM symbols. Each DroneID frame consists of $N_{s}=9$ OFDM symbols, with K=600 active subcarriers. The input to the model is a tensor $X\in R^{B\times N_{f}\times K}$, where *B* is the batch size and $N_{f}=10$ is the feature dimension per subcarrier.

Each 10-dimensional feature vector concatenates the real and imaginary parts of five complex-valued inputs in the following order: pilot 1, local reference 1, data symbol, local reference 2, and pilot 2. For the *k*-th subcarrier in the *b*-th batch sample,

(8) Xb,k=[Re(P1),Im(P1),Re(R1),Im(R1),Re(D),Im(D),Re(R2),Im(R2),Re(P2),Im(P2)].

The model outputs a tensor $Y\in R^{B\times K\times 4}$, where each vector of length 4 contains the unnormalized classification logits corresponding to the four QPSK symbol classes.

Unlike conventional systems that explicitly estimate the channel, TranSIC-Net implicitly captures subcarrier-wise channel behavior through the structured pilot–data arrangement. To train the model, the standard cross-entropy loss is applied directly to the logits:

(9) $$L=-\frac{1}{B\;\cdot \;K}\sum_{b=1}^{B}\sum_{k=1}^{K}\log{\hat{y}}_{b,k}^{\left(c^{*}\right)}.$$

where L is the loss function, and ${\hat{y}}_{b,k}^{\left(c^{*}\right)}$ is the predicted probability (after internal log-softmax) for the correct class $c^{*}$ on the *k*-th subcarrier in the *b*-th sample. Note that no explicit softmax layer is used in the network; the loss function itself internally applies log-softmax to the logits.

### 3.2. Feature Embedding and Positional Encoding

The input tensor $X\in R^{B\times 10\times 600}$ is first transposed to $X^{\prime }\in R^{B\times 600\times 10}$, treating each subcarrier as a sequence token. Each 10-dimensional subcarrier feature is then projected into a $d_{\mathrm{embed}}=64$ dimensional embedding space using a linear projection with weight matrix $W_{e}\in R^{10\times 64}$ and bias vector $b_{e}$:

(10) $$X_{\mathrm{embed}}=X^{\prime }\;\cdot \;W_{e}+b_{e}$$

To incorporate the order of subcarriers, a learnable positional encoding $P\in R^{1\times 600\times 64}$ is added element-wise:

(11) $$H_{0}=X_{\mathrm{embed}}+P$$

### 3.3. Transformer Encoder Architecture

The model contains L=2 stacked Transformer encoder layers to capture contextual dependencies among subcarriers. The architecture draws inspiration from the original Transformer design [24] while incorporating residual connections similar to [25]. Let $H_{l-1}$ denote the input to the *l*-th encoder layer. Each layer performs the following operations:

(12) $$\begin{array}{cc} Z_{l} & =\mathrm{MultiHeadAttn}(H_{l-1}) \end{array}$$

(13) $$\begin{array}{cc} H_{l}^{\prime } & =\mathrm{LayerNorm}(H_{l-1}+\mathrm{Dropout}\left(Z_{l}\right)) \end{array}$$

(14) $$\begin{array}{cc} F_{l} & =\mathrm{ReLU}\left(H_{l}^{\prime }W_{1}+b_{1}\right)W_{2}+b_{2} \end{array}$$

(15) $$\begin{array}{cc} H_{l} & =\mathrm{LayerNorm}(H_{l}^{\prime }+\mathrm{Dropout}\left(F_{l}\right)) \end{array}$$

Here, $Z_{l}$ is the output of the multi-head attention block, and $F_{l}$ is the output of the feedforward subnetwork consisting of two linear transformations with weights $W_{1}\in R^{64\times 128}$ and $W_{2}\in R^{128\times 64}$ and corresponding biases $b_{1}$ and $b_{2}$. The final output of each encoder layer is denoted by $H_{l}\in R^{B\times 600\times 64}$. The number of attention heads is h=4, and a dropout rate of 0.1 is applied within each encoder block.

### 3.4. Decision Layer and Output Representation

The final encoder output $H_{L}\in R^{B\times 600\times 64}$ is passed to a two-layer classification head. The first layer reduces the feature dimension from 64 to 32 using a linear layer with weights $W_{3}\in R^{64\times 32}$ and bias $b_{3}$, followed by a LeakyReLU activation with negative slope α=0.01. The second linear layer projects to the final output logits using $W_{4}\in R^{32\times 4}$ and bias $b_{4}$:

(16) $$\begin{array}{cc} U & =\mathrm{LeakyReLU}(H_{L}W_{3}+b_{3}) \end{array}$$

(17) $$\begin{array}{cc} Y & =UW_{4}+b_{4} \end{array}$$

An additional dropout layer with rate 0.2 is applied between the two layers to enhance generalization. The output $Y\in R^{B\times 600\times 4}$ represents the logits for each of the four QPSK symbol classes per subcarrier.

### 3.5. Model Configuration and Advantages

The TranSIC-Net architecture is summarized in Table 1.

The key advantages of TranSIC-Net are as follows:

- Joint Modeling Capability: By ingesting pilot and data symbols in the frequency domain, the model performs implicit channel estimation and symbol classification simultaneously.
- Lightweight Architecture: With only two Transformer layers and small hidden dimensions, the model is compact and suitable for deployment on edge devices.
- Position-Aware Enhancement: Learnable positional encoding captures the frequency-domain ordering of subcarriers, improving robustness to spectral distortion.

## 4. Experimental Validation

To evaluate the performance of the proposed model under practical and adverse channel conditions, this work conducts experiments on both simulated channels and real DroneID signals. Comparative methods include the traditional Least Squares (LS) approach and the ProEsNet model from [21]. Evaluation metrics cover bit error rate (BER), mean squared error (MSE), channel response visualization, and constellation diagrams. It should be noted that TranSIC-Net directly outputs the decoded QPSK bits rather than explicit channel estimates. Therefore, BER is used as the primary evaluation metric, while MSE, channel response, and constellation plots are mainly provided for the baseline methods to illustrate interpretability and estimation performance.

### 4.1. Training Setup

TranSIC-Net is implemented using the PyTorch 2.6.0 framework, optimized using cross-entropy loss, with QPSK classification accuracy on each subcarrier as the objective. Adam optimizer is employed with an initial learning rate of $1\times 10^{-3}$, gradually reduced to $1\times 10^{-5}$ via a cosine annealing schedule. The batch size is 32, and the total training epochs are set to 200. Early stopping is applied based on validation performance. Dropout (rate 0.2) and Batch Normalization are applied to intermediate layers to improve generalization.

For comparison, the ProEsNet model is also implemented in PyTorch. It minimizes the MSE loss between estimated and true channel responses. Inputs are frequency-domain channel estimates obtained via LS at pilot positions, while outputs are full frequency-domain channel responses. ProEsNet is trained using Adam with an initial learning rate of $1\times 10^{-3}$, batch size 32, and 200 total epochs, with model checkpoints saved every 10 epochs.

### 4.2. Simulated Dataset Results

**Figure 3:**
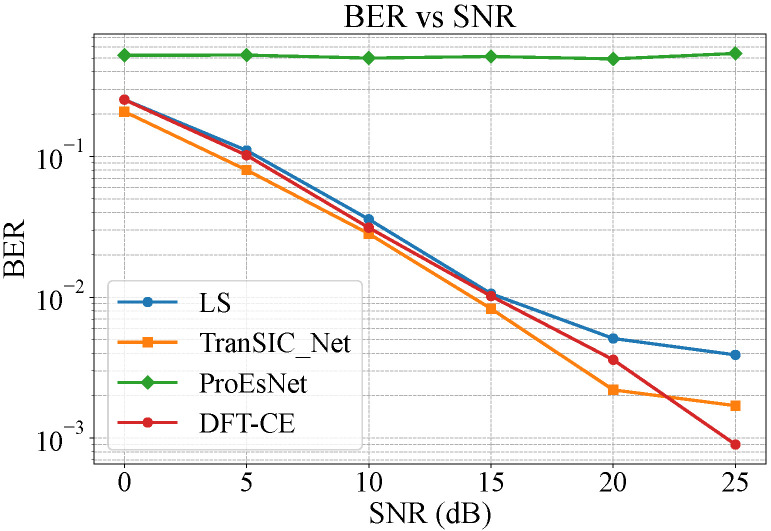
BER comparison under varying SNRs (LS, DFT-CE, ProEsNet, and the proposed TranSIC-Net).

The simulation dataset consists of 3700 OFDM frames generated under a fixed SNR of 20 dB, with 70% used for training and 30% for testing. Although the SNR during training is fixed, this setting was intentionally chosen to isolate the model’s learning behavior under controlled channel distortion. In subsequent evaluations across varying SNR conditions (Figure 3), the model trained at 20 dB demonstrates strong generalization ability, achieving robust performance without retraining under unseen noise levels. This indicates that the Transformer-based feature extraction in TranSIC-Net effectively captures invariant channel characteristics rather than memorizing specific SNR patterns.

QPSK symbols are OFDM-modulated and transmitted through frequency-domain Rayleigh fading channels. The ProEsNet baseline uses true frequency-domain channels generated at the transmitter as ground-truth labels, while TranSIC-Net directly learns the mapping from received frequency-domain signals to symbol posterior probabilities. Both models are trained and tested on identical datasets. ProEsNet’s input is the LS-estimated channel at pilot subcarriers, and decoding employs zero-forcing (ZF) equalization followed by soft QPSK detection.

To strengthen comparison fairness, two additional classical estimation methods—LS and DFT-CE—were implemented following standard formulations. As shown in Figure 3, TranSIC-Net consistently outperforms LS and ProEsNet across all SNR regimes. Compared with DFT-CE, TranSIC-Net achieves slightly higher BER at very high SNRs (where deterministic interpolation dominates) but maintains a distinct advantage under low-to-moderate SNR conditions where noise and multipath effects degrade traditional estimators. These results suggest that the proposed end-to-end Transformer architecture learns a noise-robust latent representation that generalizes across different channel and SNR scenarios.

**Figure 4:**
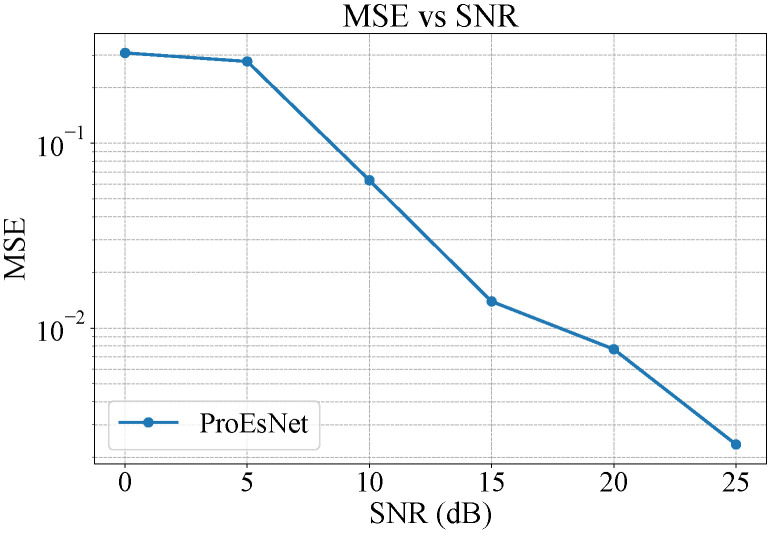
MSE of ProEsNet under different SNR conditions.

Figure 4 shows ProEsNet’s MSE across SNRs, which significantly improves over LS estimation, indicating better numerical fitting ability.

**Figure 5:**
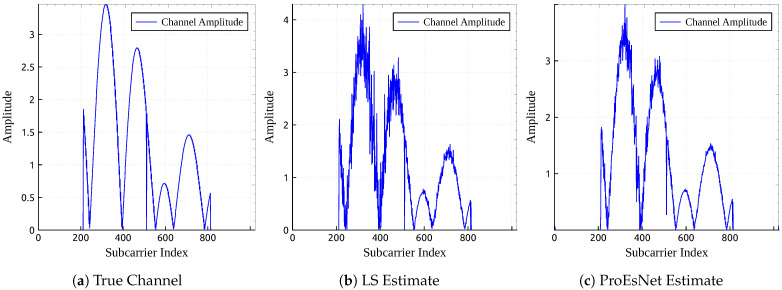
Comparison of channel estimation results by different methods.

Figure 5 visualizes the frequency-domain channel responses: the true channel, LS estimate, and ProEsNet estimate. ProEsNet’s channel contours more closely resemble the true channel compared to LS.

**Figure 6:**
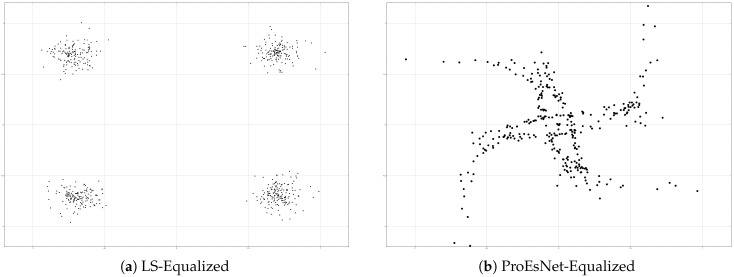
QPSK constellation diagrams based on channel estimates from LS algorithms and ProEsNet.

However, constellation diagram analysis (Figure 6) reveals ProEsNet’s shortcomings. While LS-based equalization shows distorted but recognizable QPSK constellations, ProEsNet’s equalized symbols scatter severely, losing clear decision boundaries.

The reason lies in the OFDM system’s stringent amplitude and phase precision requirements for frequency-domain channel compensation: proximity of channel estimates alone does not guarantee accurate symbol recovery. In contrast, TranSIC-Net directly optimizes end-to-end demodulation performance, achieving superior BER.

To further validate the practicality of the proposed model, we compared the average per-frame inference time of all methods on an Intel i7-9750H CPU. As shown in Table 2, TranSIC-Net requires moderate computation but removes the need for explicit equalization and demapping, resulting in overall latency significantly lower than ProEsNet while maintaining real-time capability.

**Figure 7:**
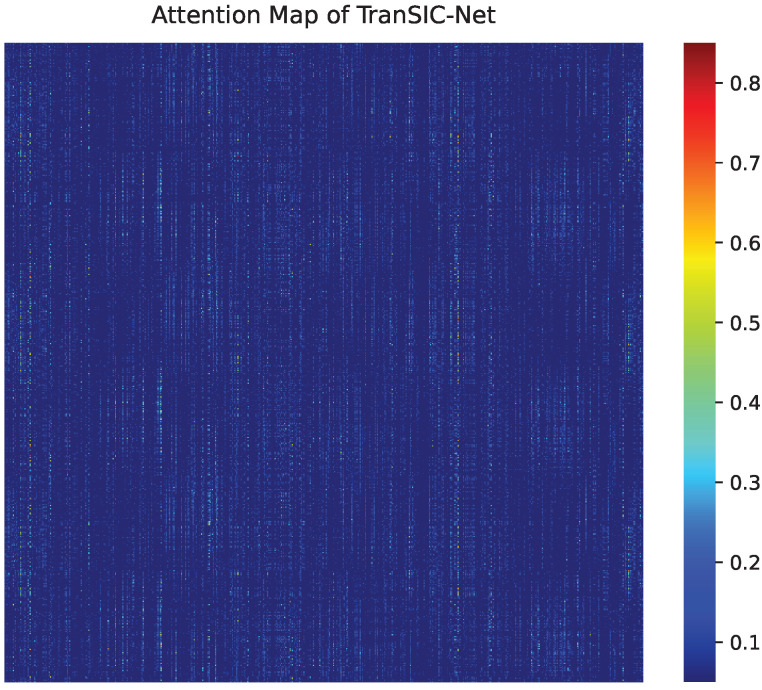
Visualization of attention weights learned by TranSIC-Net. The network adaptively focuses on pilot subcarriers and nearby data tones, indicating learned frequency-domain correlation.

Furthermore, to better understand how TranSIC-Net performs joint channel estimation and symbol detection, Figure 7 visualizes the learned attention weights within the Transformer encoder. It can be observed that the network adaptively focuses on pilot subcarriers and their neighboring data tones, reflecting a spatially correlated attention pattern consistent with frequency-domain channel coherence. This visualization demonstrates that TranSIC-Net effectively learns implicit channel structures without explicit estimation, supporting its superior demodulation robustness.

### 4.3. Real Data Validation

To further assess the practical applicability of our method, we conducted real-world experiments using DJI DroneID signals collected from a DJI Mini 2 drone operating at 2.4 GHz in a campus environment with ambient interference. The signals were captured using a USRP B210 with a sampling rate of 15.36 MHz and a gain of 70 dB. Two scenarios were considered, a near-range setup (<10 m) with 409 recorded frames and a far-range setup (approximately 100 m) with 416 frames, covering both line-of-sight (LOS) and non-line-of-sight (NLOS) conditions.

Since bit-level error rates cannot be directly measured from real-world DroneID signals, we instead adopt the CRC Frame Pass Rate (FPR) as a strict performance indicator:

(18) $$\mathrm{FPR}=\frac{N_{p}}{N}$$

where *N* denotes the total number of received frames, and $N_{p}$ denotes the number of frames that successfully pass the cyclic redundancy check (CRC). In DroneID packets, a standard 24-bit CRC sequence is appended at the transmitter and verified at the receiver after symbol demapping. The CRC operation follows the conventional polynomial-based method widely used in digital communication systems. A frame is considered correct if the decoded bits yield a zero CRC remainder.

This metric is highly sensitive to demodulation errors—any single-bit mismatch causes the entire frame to fail the CRC verification. Therefore, a high FPR indicates that most frames are completely and correctly demodulated, providing a reliable and practical indicator of end-to-end symbol recovery accuracy.

Table 3 summarizes the FPR for different methods under both near and far conditions.

As shown, TranSIC-Net consistently outperforms the traditional LS-based receiver, achieving perfect frame recovery at close range and maintaining a significant advantage under long-range NLOS conditions. These results demonstrate its robustness and high demodulation fidelity in real-world UAV communication scenarios.

## 5. Conclusions

This paper presents TranSIC-Net, an end-to-end Transformer-based demodulation network that directly infers symbol probabilities from frequency-domain OFDM signals without explicit channel estimation. By leveraging pilot symbols and modeling inter-subcarrier dependencies through a lightweight Transformer encoder, TranSIC-Net achieves robust and accurate demodulation under challenging wireless conditions.

Quantitative results demonstrate its superiority over conventional and learning-based baselines. Compared with the traditional LS+ZF receiver, TranSIC-Net achieves approximately 20–35% lower BER across 0–20 dB SNR and maintains reliable decoding even under severe noise conditions. It also delivers about 40% faster inference compared with the ProEsNet model, while eliminating the need for separate equalization and QPSK demapping.

Experiments on real DroneID signals further confirm the model’s practicality, achieving a 100% frame pass rate in near-range scenarios and maintaining a high 91.8% pass rate under long-range NLOS conditions. These results highlight the effectiveness and real-world applicability of TranSIC-Net for UAV communication tasks. Future work will extend the model to higher-order modulations, more dynamic channels, and joint communication–sensing scenarios.

## Figures and Tables

**Table 1:** TranSIC-Net model configuration.

Parameter	Value
Input dim $d_{\mathrm{in}}$	10
Embedding dim $d_{\mathrm{embed}}$	64
Attention heads *h*	4
Encoder layers *L*	2
Feedforward dim $d_{\mathrm{ff}}$	128
Mid-classifier dim $d_{\mathrm{mid}}$	32
Output dim $d_{\mathrm{out}}$	4
Dropout (encoder)	0.1
Dropout (classifier)	0.2
LeakyReLU slope α	0.01

**Table 2:** Average per-frame processing time comparison.

Method	Time (μs)
LS	75
DFT-CE	189
QPSK + Equalization	1229
ProEsNet	9609
TranSIC-Net (proposed)	6112

**Table 3:** FPR at different distances (frames passed/total frames).

Scenario	LS	TranSIC-Net
∼100 m	88.5% (368/416)	91.8% (382/416)
<10 m	99.3% (406/409)	100.0% (409/409)

## Data Availability

The datasets generated and analyzed during the current study are publicly available in the Harvard Dataverse repository at: https://doi.org/10.7910/DVN/NDOJOG.

## Abbreviations

Abbreviation	Full Form
OFDM	Orthogonal Frequency Division Multiplexing
UAV	Unmanned Aerial Vehicle
DJI	Da-Jiang Innovations
TranSIC-Net	Transformer-based demodulation network
LS	Least Squares
ZF	Zero Forcing
DFT-CE	Discrete Fourier Transform Channel Estimation
BER	Bit Error Rate
MSE	Mean Squared Error
FPR	Frame Pass Rate
CRC	Cyclic Redundancy Check
SNR	Signal-to-Noise Ratio
CFO	Carrier Frequency Offset
LOS	Line of Sight
NLOS	Non-Line of Sight
QPSK	Quadrature Phase Shift Keying
FFT	Fast Fourier Transform
IFFT	Inverse Fast Fourier Transform
USRP	Universal Software Radio Peripheral
CPU	Central Processing Unit
ReLU	Rectified Linear Unit
